# Brain morphometry in transient global amnesia: a triangulated analysis approach

**DOI:** 10.1590/1980-5764-DN-2024-0163

**Published:** 2024-11-11

**Authors:** Micaela Anahi Hernández, Hernán Chaves, Ricardo Francisco Allegri, Ismael Luis Calandri

**Affiliations:** 1 Fleni, Department of Cognitive Neurology, Buenos Aires, Argentina.; 2 Fleni, Diagnostic Imaging Department, Buenos Aires, Argentina.; 3 Vrije Universiteit Amsterdam, Amsterdam Neuroscience, Alzheimer Center Amsterdam, Department of Neurology, Amsterdam, the Netherlands

**Keywords:** Amnesia, Transient Global, Magnetic Resonance Imaging, Amnésia Global Transitória, Imageamento por Ressonância Magnética

## Abstract

Transient global amnesia (TGA) is a rare, temporary, sudden-onset disturbance in anterograde episodic memory with unclear pathophysiology. Previous brain volumetric analysis in TGA patients showed varied results.

**Objective:**

To explore brain morphometry, hypothesizing that patients with TGA exhibit structural alterations.

**Methods:**

A case-control study was performed involving TGA subjects (n=50) and matched healthy controls (n=50). Both groups underwent a 3D-T1 weighted structural MRI on a 3T scanner, and voxel-based morphometry (VBM), region-based morphometry (RBM), and surface-based morphometry (SBM) were analyzed.

**Results:**

After performing the VBM, RBM, and SBM analyses, no consistent and statistically significant differences were found after applying multiple corrections.

**Conclusion:**

Despite previous studies showing volumetric changes in TGA patients, our results differ from this. The discrepancy could be due to sample size and timing of MRI scans. While our findings do not explain TGA pathophysiology, they support a network dysfunction as a possible mechanism and discards a structural alteration as a predisposing factor for TGA.

## RESUMO

Amnésia global transitória (AGT) é uma alteração rara, temporária e de início súbito na memória episódica anterógrada, com uma fisiopatologia pouco clara. Análises volumétricas cerebrais anteriores em pacientes com AGT apresentaram resultados variados.

**Objetivo:**

Explorar a morfometria cerebral, com a hipótese de que pacientes com AGT exibam alterações estruturais.

**Métodos:**

Realizou-se um estudo de caso-controle envolvendo indivíduos com AGT (n=50) e controles saudáveis (n=50). Os dois grupos foram submetidos a uma ressonância magnética ponderada em 3D-T1 em um *scanner* de 3T, e analisou-se a morfometria baseada em *voxel* (MBV), morfometria baseada em região (MBR) e morfometria baseada em superfície (MBS).

**Resultados:**

Após realizar as análises de MBV, MBR e MBS, não foram observadas diferenças consistentes e estatisticamente significativas após a aplicação de correções múltiplas.

**Conclusão:**

Apesar de estudos anteriores mostrarem alterações volumétricas em pacientes com AGT, os resultados deste estudo diferem disso. A discrepância pode ser atribuída ao tamanho da amostra e ao momento em que as ressonâncias foram realizadas. Embora os presentes achados não expliquem a fisiopatologia da AGT, eles apoiam a disfunção de rede como um possível mecanismo e descartam uma alteração estrutural como fator predisponente para AGT.

## INTRODUCTION

Transient global amnesia (TGA) is an episodic memory disorder affecting middle-aged and older adults^1^, characterized by difficulty in acquiring new information (anterograde amnesia) and recalling recent events preceding the episode (retrograde amnesia)^2^.

Involvement of the hippocampus can be considered the structural correlate of the amnestic deficit. The region of the hippocampus mostly affected is the cornu ammonis CA1 subfield, reported to have a particular vulnerability to metabolic stress and cytotoxic damage, often presenting with a restrictive lesion in diffusion-weighted magnetic resonance imaging (MRI) sequences that becomes visible 24–48 hours after the episode and may persist for 7–10 days^3^.

Nevertheless, Single Photon Emission Computed Tomography (SPECT)^4^ and network^5^ studies described other regions involved in TGA, beyond the hippocampus, including the cingulate gyrus, the anterior thalamic nucleus, the putamen and caudate nucleus, the corpus callosum and fornix, among others.

The underlying pathophysiological mechanism of TGA remains uncertain, encompassing potential causes such as isolated arterial ischemia, venous congestion linked to mesial temporal ischemia, mechanisms related to epilepsy, migraines, cortical spreading depression, and psychogenic factors^3^.

Despite this, TGA generally carries a favorable prognosis, as patients fully recover within 24 hours. However, some individuals may report lingering cognitive deficits even after the resolution of acute episodes^6,7,8,9^.

The morphometric characteristics of cortical structures and hippocampal subfields in individuals with TGA have been examined in prior studies, yielding inconsistent findings^10,11,12,13^.

Considering that although the brain region expected to be most affected is the hippocampus, other regions were also described to be involved. Our hypothesis is that individuals diagnosed with TGA might exhibit wide structural alterations. Therefore, we decided to conduct a comprehensive analysis of brain structures in subjects after a TGA episode, comparing them to a sample of healthy controls. To scrutinize local variations and subtle changes in gray matter, voxel-based morphometry (VBM) was employed for the analysis of cerebral structure. Additionally, region-based morphometry (RBM) was utilized with a specific focus on hippocampal structures, and surface-based morphology (SBM) to investigate potential alterations in cortical thickness and surface area.

## METHODS

### Subjects

Participants who experienced a TGA event were enrolled, identified, and recruited from the emergency department of a neurological tertiary center in Buenos Aires, Argentina, spanning the period between December 2019 and June 2022. The events were defined according to the Hodges and Warlow diagnostic criteria^14^:

- anterograde amnesia witnessed by an observer, with symptoms resolving within 24 hours;
- cognitive impairment confined to amnesia, without clouding of consciousness, loss of personal identity, focal neurological or epileptic signs, or recent head trauma or seizures; and
- absence of structural brain lesions.

To ensure accurate diagnoses, all subjects underwent neurological examination and detailed assessment to rule out differential diagnoses, including an electroencephalogram, jugular venous doppler with reflux measurement, and an on-call structural brain MRI, which specifically included a diffusion-weighted sequence.

To avoid confounders, the 3D-T1 weighted structural MRI was performed and analyzed at least ten days after the event. To facilitate patient recruitment and ensure optimal adherence, a maximum limit of three months was established for performing MRI scans.

### Control group

The control group comprised 50 individuals matched for age and gender, who consulted at the Memory Clinic, all of whom had cognitive complaints and underwent brain MRI and routine cognitive assessments for this reason. Normality among participants was defined by the absence of any history of neurological disorders, the absence of abnormal findings in MRI scans as evaluated by a trained neuroradiologist, and a performance score higher than -1.5 Z score (adjusted for age, gender, and education) for all neurocognitive tests included in the Uniform Data Set version 3 (UDS3) neuropsychological battery^15^.

### Magnetic resonance imaging acquisition and conversion

MRI scans were acquired using a GE Discovery 750 3T scanner with a 32-channel head coil. 3D T1-weighted images parameters are summarized in Table 1.

**Table 1:** 3D Inversion time-weighted sequence parameters.

Parameter	Value
NEX	1
Matrix	256 x 256
Slice thickness	1.1 mm
Field of view	230 mm
Voxel size	1.1 x 1.1 x 1.1 mm
TR	8.26 ms
TE	3.26 ms
TI	450 ms

Abbreviations: NEX, Number of excitations; TR, Repetition time; TE, Echo time; TI, Inversion time.

Images were downloaded from our institutional Picture Archiving and Communication Systems (PACS), anonymized, and converted to NifTI format using the dcm2niix module form MRIcroGL 1.2.2.

### Processing

NifTI-converted T1-weighted images were normalized to a template space and segmented into gray matter (GM), white matter (WM), and cerebrospinal fluid (CSF). Spatial registration was performed using the default CAT12 approach (geodesic shooting). The cortical thickness of GM segmentations was calculated using the standard CAT12 pipeline. After segmentation and parcellation, volume and surface data were smoothed with 8- and 15-mm kernels, respectively.

VBM, RBM, and SBM analyses were performed on each MRI, based on the Cobra^16^ and Neuromorphometrics^17^ atlases, to ensure that the results were consistent across the different analysis methods. VBM, RBM, and SBM steps, as well as quality check and statistical analyses, were performed using Computational Anatomy Toolbox 12 (CAT12)^18^, an extension to Statistical Parametrical Mapping (SPM12) software package^19^.

VBM overlays were warped to a template made from the averaged 100 brains included in this study, while RBM were warped to the MNI 2009c NLIN asymmetric T2 template.

### Quality check

Input images and preprocessing quality were checked using the quality assurance reports generated by CAT12. Image resolution, noise, and bias were graded and summarized in a weighted average grade (WAG) by CAT12. WAG is defined as following:

1. Excellent,
2. Good,
3. Satisfactory,
4. Sufficient,
5. Critical, and
6. Unacceptable/failed.

WAG from most open-access databases typically ranges between 2 and 3. Additionally, all input images were visually inspected by an experienced neuroradiologist (HC).

Total intracranial volume (TIV) as well as absolute and relative CSF, GM, and WM volumes were estimated. A global thickness estimation was also recorded in the quality assurance report. To check whether the normalization and spatial registration was successful across all subjects, a single slice display of the GM segmentation masks was generated and visually inspected. Lastly, the homogeneity of the sample was examined by generating a correlation matrix and boxplot. Processing time was recorded in minutes.

### Group-level analysis

For VBM and RBM, an independent two-sample t-test with TIV, gender, and age as covariates was modeled and estimated. VBM was implemented without Jacobian modulation of the intensities or global scaling. For SBM, an independent two-sample t-test without covariates was modeled and estimated. A p<0.05, corrected for multiple comparisons applying the Holm-Bonferroni test, was considered statistically significant.

## RESULTS

### Subjects

A total of 100 subjects were included, with 50 in the TGA group and 50 in the healthy control group. Fifty-four percent of the subjects were female. Mean age was 67 years old (range 57–77). Regarding cognitive results, the Montreal Cognitive Assessment (MoCA) showed no statistically significant results between TGA and control patients. Demographic characteristics of TGA and control group are summarized in Table 2.

**Table 2:** Demographic characteristics.

		TGA (n=50)	Control (n=50)	p-value*	q-value^†^
Age (mean, SD)		67 (10)	68 (6)	>0.9	>0.9
Sex	Female (n, %)	27 (54)	28 (56)	0.8	
	Male (n, %)	23 (46)	22 (44)		
Education level		14.8 (3.5)	14.6 (3.4)	0.6	>0.9
MoCA (Z score) (mean, SD)		0.16 (0.88)	0.49 (0.52)	0.13	0.5
TGA recurrence (n, %)		7 (14%)	NA		
CA1 diffusion lesion (n, %)		10 (20%)	NA		

Abbreviations: TGA, transient global amnesia; SD, standard deviation; MoCA, Montreal Cognitive Assessment; CA1, *Cornus ammonis* 1; NA, not applicable.

Notes: *Wilcoxon rank sum test; Pearson’s χ^2^ test;

^†^False discovery rate correction for multiple testing.

Gross morphometric data (TIV, GM, WM, and CSF volumes) are summarized in Table 3.

**Table 3:** Gross volumetric data summarized by group.

	TGA (n=50)	HC (n=50)	p-value
Mean TIV±SD	1,486±143 mL	1,466±120 mL	0.482
Mean GM volume±SD	623±53 mL	608±46 mL	0.141
Mean WM volume±SD	494±63 mL	480±65 mL	0.301
Mean CSF volume±SD	396±85 mL	378±74 mL	0.595

Abbreviations: TGA, transient global amnesia; HC, healthy controls; TIV, total intracranial volume; SD, standard deviation; GM, grey matter; WM, white matter; CSF, cerebrospinal fluid.

### Magnetic resonance imaging quality control

Input 3D T1- weighted images had a mean WAG of 2.5 (range 1.8–5.3), which is defined as good. Only two subjects had critical quality images (with values higher than or equal to 5), and no subjects had unacceptable quality images (higher than or equal to 6). Mean processing time was 32±4 minutes.

### Voxel-based morphometry

**Figure 1:**
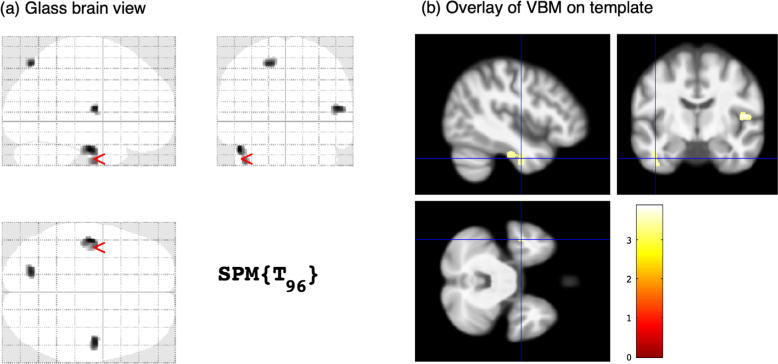
Voxel-based morphometry results. Regions with reduced grey matter in subjects with transient global amnesia compared to controls, uncorrected p<0.001, 80 voxels cluster threshold. (A) Glass brain maximum intensity projection view showing 3 suprathreshold clusters with peak coordinates at left fusiform gyrus, right precentral gyrus (opercular), and left superior parietal lobe. (B) Corresponding overlay of thresholder Statistical Parametrical Mapping on average template, with cross-hairs at the left fusiform gyrus cluster.

Using a p<0.001 and a minimum threshold of 80 voxels cluster (88 mm^3^), three regions with reduced GM were identified in subjects with TGA compared to controls, with peak coordinates at the left fusiform gyrus, right opercular precentral gyrus, and left superior parietal lobe (Figure 1). However, no suprathreshold clusters were found using a p<0.05 corrected for multiple comparisons.

### Region-based morphometry

**Figure 2:**
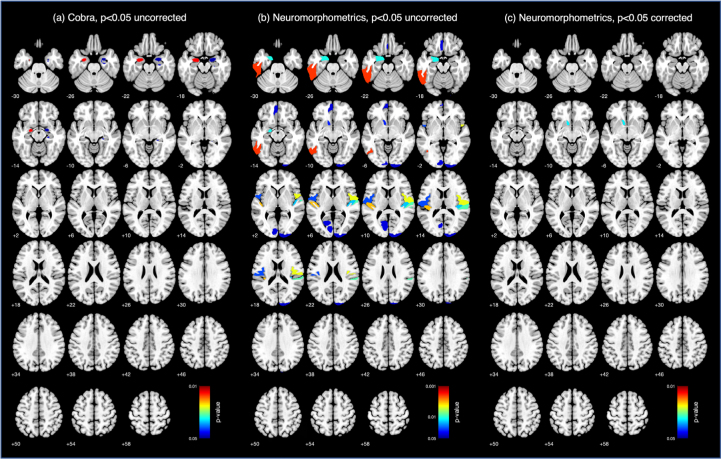
Region-based morphometry results. Group comparisons showing decreased volume in the transient global amnesia group compared to controls using uncorrected p<0.05 in the Cobra (A) and Neuromorphometrics (B).

Using an uncorrected p<0.05, decreased GM volume was observed in cortical and subcortical regions of interest (ROI) in the TGA group compared to controls. However, only the volume difference in the left accumbens nucleus on the Neuromorphometrics atlas subsisted after applying the Holm-Bonferroni correction (Figure 2).

### Surface-based morphometry

**Figure 3:**
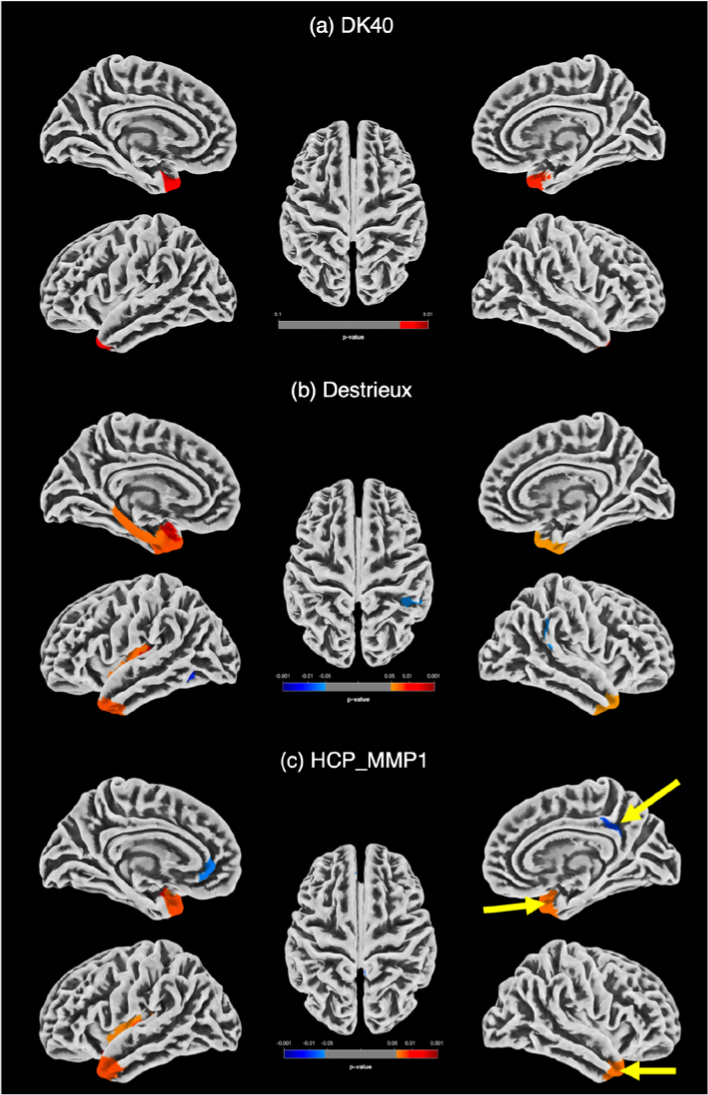
Surface based morphometry results. Group comparisons showing decreased cortical thickness (orange-red) in the transient global amnesia group compared to controls (uncorrected p<0.05) in the DK40 (A), Destrieux (B), and HCP MMP 1.0 (C) atlases. Only the right temporal pole and ventromedial visual area 2 differences subsisted after Holm-Bonferroni corrected p<0.05 on the HCP atlas (arrows in C).

Using an uncorrected p<0.05, decreased cortical thickness was found in the TGA group compared to controls in the DK40, Destrieux, and HCP MMP 1.0 atlases, mainly in the temporal poles. However, only the cortical thickness differences observed in the right temporal pole and ventromedial visual area 2 on the HCP atlas subsisted after applying Holm-Bonferroni correction (Figure 3).

## DISCUSSION

The objective of this study was to investigate brain cortical structure in individuals with a history of TGA in comparison to a control group. Utilizing VBM, RBM, and SBM analyses, and after applying multiple corrections, the findings revealed no consistent and statistically significant differences in gray matter morphometry.

This study encompasses a relatively large sample of individuals with TGA, where three distinct types of analyses were conducted. The examination of cortical volume enables an exploration of brain structure, acknowledging that cortical volume integrates both surface area and thickness. VBM faces challenges in accurately measuring cortical morphology. Surface-based analysis (SBA) offers enhanced accuracy in representing cortical geometry and analyzing cortical shape and folding patterns, which are not attainable through VBM. The hippocampus plays a crucial role in memory, especially in episodic memory. Region-based analysis allowed for a targeted regional investigation with a focus on the hippocampal region.

Despite the findings across all three analytical approaches, the results deviate from those reported in previous works. For instance, Pirlich et al.^11^ reported an increase in GM volume within the bilateral hippocampus. In contrast, Park et al.^10^ observed a significant decrease in GM volume in the left hippocampus, left amygdala, bilateral cingulum, and the right posterior lobe of the cerebellum in TGA patients compared to control subjects. Hodel et al.^13^ reported lower cortical thickness compared with controls in the orbitofrontal, cingulate, and inferior temporal cortices. Additionally, Kim et al.^12^ identified widespread alterations, involving regions with reduced and increased cortical thickness across both cerebral hemispheres. These results are attributed to the relatively large sample size, which includes a great number of patients compared to previous works. Furthermore, the decision to conduct brain structural MRI in the subacute stage contributes to the elimination of potential confounding factors.

Cortical spreading depression (CSD) is one of the most widely recognized explanations for the pathogenesis of TGA^4^. Through a mechanism of glutamate-mediated neuronal and glial depolarization, CSD causes transient hypoxia and metabolic stress that might affect the CA1 hippocampal subfield. This mechanism has the potential to disrupt the normal functioning of neural networks in the brain, altering the synchrony and connectivity between brain areas^4^.

Previous studies showed cortical volume reduction in default mode network (DMN) regions and cerebellum in TGA patients compared to controls^10,12,20^. Various functional MRI studies suggest the engagement of the episodic memory network^21^, the executive network^22^, and the limbic network^23^ in TGA episodes.

While this study’s results do not offer insights into the pathophysiology, the absence of asymmetries in our triadic approach analysis supports network dysfunction as a possible mechanism underlying the clinical findings and dismisses a structural cause as a predisposing factor for the occurrence of an episode.

The present study has certain limitations. If the volume reduction is very subtle, it could explain the lack of statistical significance in our results. Conducting a study with a larger sample size would likely provide more insight into this aspect. Besides, as TGA is a multifactorial condition, cortical volume may not be the primary marker of structural changes in TGA and other imaging modalities may be more informative in early stages. Because the syndrome is infrequent, patients are unlikely to have a brain MRI scan before the event that would allow comparison with the current one to determine if there is a volume variation in the patient.

In conclusion, no significant gray matter asymmetries were found in TGA patients compared to controls after VBM, RBM, and SBM analyses. The main contribution of our work is that it was performed on a relatively large sample of TGA patients using three different morphometric analyses, demonstrating that our results are consistent.
